# Cognitive and Neuropsychiatric Manifestations of COVID-19 and Effects on Elderly Individuals With Dementia

**DOI:** 10.3389/fnagi.2020.588872

**Published:** 2020-10-26

**Authors:** Silvia Alonso-Lana, Marta Marquié, Agustín Ruiz, Mercè Boada

**Affiliations:** ^1^Research Center and Memory Clinic, Fundació ACE, Institut Català de Neurociències Aplicades, Universitat Internacional de Catalunya, Barcelona, Spain; ^2^Networking Research Center on Neurodegenerative Diseases (CIBERNED), Instituto de Salud Carlos III, Madrid, Spain

**Keywords:** COVID-19, SARS-CoV-2, pandemics, cognition, neuropsychiatry, dementia, Alzheimer

## Abstract

The coronavirus disease 2019 (COVID-19) caused by the severe acute respiratory syndrome coronavirus 2 (SARS-CoV-2) has rapidly spread worldwide and has had unprecedented effects in healthcare systems, economies and society. COVID-19 clinical presentation primarily affects the respiratory system causing bilateral pneumonia, but it is increasingly being recognized as a systemic disease, with neurologic manifestations reported in patients with mild symptoms but, most frequently, in those in a severe condition. Elderly individuals are at high risk of developing severe forms of COVID-19 due to factors associated with aging and a higher prevalence of medical comorbidities and, therefore, they are more vulnerable to possible lasting neuropsychiatric and cognitive impairments. Several reports have described insomnia, depressed mood, anxiety, post-traumatic stress disorder and cognitive impairment in a proportion of patients after discharge from the hospital. The potential mechanisms underlying these symptoms are not fully understood but are probably multifactorial, involving direct neurotrophic effect of SARS-CoV-2, consequences of long intensive care unit stays, the use of mechanical ventilation and sedative drugs, brain hypoxia, systemic inflammation, secondary effects of medications used to treat COVID-19 and dysfunction of peripheral organs. Chronic diseases such as dementia are a particular concern not only because they are associated with higher rates of hospitalization and mortality but also because COVID-19 further exacerbates the vulnerability of those with cognitive impairment. In patients with dementia, COVID-19 frequently has an atypical presentation with mental status changes complicating the early identification of cases. COVID-19 has had a dramatical impact in long-term care facilities, where rates of infection and mortality have been very high. Community measures implemented to slow the spread of the virus have forced to social distancing and cancelation of cognitive stimulation programs, which may have contributed to generate loneliness, behavioral symptoms and worsening of cognition in patients with dementia. COVID-19 has impacted the functioning of Memory Clinics, research programs and clinical trials in the Alzheimer’s field, triggering the implementation of telemedicine. COVID-19 survivors should be periodically evaluated with comprehensive cognitive and neuropsychiatric assessments, and specific mental health and cognitive rehabilitation programs should be provided for those suffering long-term cognitive and psychiatric sequelae.

## Introduction

In late December 2019, China reported a cluster of cases of pneumonia caused by a novel coronavirus, the severe acute respiratory syndrome coronavirus 2 (SARS-CoV-2), responsible for the subsequently named coronavirus disease 2019 (COVID-19). Since then, positive cases and deaths rapidly began to rise and spread worldwide, and the World Health Organization (WHO) declared the outbreak as a pandemic on 11 March 2020. Currently, as of 27 September 2020, the WHO has reported more than 32 million laboratory-confirmed positive cases and 991,224 deaths for COVID- 19 worldwide, half of them in the Americas followed by Europe (World Health Organization, 2020).

SARS-CoV-2 infection primarily affects the respiratory system, being fever and cough two of the most common acute symptoms in those symptomatic (Borges Do Nascimento et al., 2020; Lechien et al., 2020), but around 20% of individuals can suffer a more severe disease with critical and life-threatening respiratory complications (Wu and McGoogan, 2020). Besides, COVID-19 is increasingly being recognized as a systemic disease, and multiple neurologic manifestations have been reported in around 35.6% of cases (Tsai et al., 2020). They encompass non-specific symptoms, mainly headache and myalgia, along with hyposmia and dysgeusia, but there is also evidence of more severe complications such as neuroinflammatory syndromes, encephalopathies, ischemic strokes or Guillain-Barré syndromes, that have also been described in relation to other respiratory viruses (Ellul et al., 2020; Paterson et al., 2020; Roman et al., 2020; Tsai et al., 2020). Severely ill patients present more frequently these neurologic manifestations (Pinzon et al., 2020). In a large cohort of 841 patients hospitalized due to COVID-19 from two clinical centers in Spain, neurological symptoms were present in 54.7% of cases, a rate that increased up to 64.7% in those with a severe infection (Romero-Sanchez et al., 2020). Altered levels of consciousness were the most common neurologic manifestation in this group (Romero-Sanchez et al., 2020). Moreover, these mild disorders of consciousness along with focal neurologic deficits were the reason for the initial consultation in 2.5% of the patients and neurological complication were the main cause of death in 4.1% of the total deceased patients (Romero-Sanchez et al., 2020).

Although COVID-19 may affect individuals from all ages, elderly population is disproportionately impacted by the pandemic. Hospitalization and mortality rates increase drastically after 65 years of age (Centers for Disease Control and Prevention, 2020; Goujon et al., 2020) and current evidence points to age along with male sex and the presence of comorbid medical conditions as factors of poor prognosis and higher risk of death (Lu et al., 2020; Martin-Sanchez et al., 2020; Williamson et al., 2020). Moreover, elderly people with chronic diseases such as dementia often present atypical symptoms at onset of COVID-19 such as altered mental status (including confusion, agitation, disorientation, refusal of care, disorientation, and loss of appetite) (Bianchetti et al., 2020; Isaia et al., 2020; Ward et al., 2020). This atypical presentation may delay appropriate diagnosis and treatment and consequently, it may worsen their prognosis and survival.

Thus, elderly population is not only more likely to suffer a more severe illness from COVID-19 but also, they are more vulnerable to possible persisting health consequences. Long-term complications in those patients who have survived are currently unknown. However, as it has been seen in similar viral infection and survivors of critical illness, some of these patients might show neurological sequelae in the next months and years in form of lasting neuropsychiatric and cognitive impairment (Lam et al., 2009; Desai et al., 2011; Herridge et al., 2016; Troyer et al., 2020). Therefore, in this study we review the evidence regarding the neuropsychiatric and cognitive manifestations of COVID-19 as well as its direct and indirect consequences in survivors, especially in elderly individuals with dementia.

## Neuropsychiatric Manifestations of COVID-19

Manifestations such as insomnia, anxiety, post-traumatic stress symptoms (PTSD), psychosis and mood disorders have been described in several reports (see Table 1; Dinakaran et al., 2020; Liguori et al., 2020; Nalleballe et al., 2020; Rogers et al., 2020; Romero-Sanchez et al., 2020; Vindegaard and Benros, 2020). A study that retrieved data from a global health collaborative platform that included medical records of 40,469 COVID-19 positive cases, mostly from the United States (US) (76%), found that 22.5% had neurological and/or psychiatric manifestations, being anxiety and related disorders the most prevalent (4.6%) (Nalleballe et al., 2020).

**TABLE 1:** Neuropsychiatric manifestations of COVID-19.

Study	Type of COVID-19 sample	Country	N	Sex (M/F)	Age mean (SD)	Type of assessment	Results
Cai et al., 2020	Cured COVID-19 patients in quarantine after discharge from hospital	China	126	60/66	45.7 (14.0)	Online questionnaire consisting of self-report scales: – Post-traumatic stress disorder self-rating scale (PTSD-SS) – Self-rating depression scale (SDS) – Self-rating anxiety scale (SAS)	Percentage of subjects who met the cut-off value of the scale: – Overall: 54.8% – Depression: 38.1% – PTSD: 31% – Anxiety: 22.2% – Anxiety and depression: 11.9%
Dinakaran et al., 2020	Review of 12 studies: – 9 case report and series – 1 observational study – 2 case control studies	Global: China: 4 studies US: 3 studies Others: France, Japan, Saudi Arabia, Spain and Perú	–	–	–	–	Evidence of neuropsychiatric manifestations: – Delirium: 4 studies – Psychosis: 2 studies – Mood swings: 1 study – Increased psychological distress in individuals with pre-existing epilepsy and psychiatric disorders: 2 studies
Helms et al., 2020	Patients admitted to an ICU with ARDS due to COVID-19	France	58	–	63 (median)	Confusion Assessment Method for the ICU (CAM-ICU)	Prevalence of symptoms: – Positive CAM-ICU: 65% – Agitation: 69%
Liguori et al., 2020	Hospitalized patients due to COVID-19	Italy	103	59/44	55 (14.65)	Anamnestic interview	Prevalence of symptoms: – Sleep impairment: 49.51% – Depression: 37.86% – Anxiety: 33.01% – Confusion: 22.33%
Nalleballe et al., 2020	Data retrieved from a platform that included medical records of COVID-19 positive cases	Global (76% US)	40,469	18,364/22,063	–	ICD-10 diagnosis for neurological and psychiatric symptoms during or within 1 month after COVID-19 diagnosis	Prevalence of manifestations: – Overall: 22.5% – Anxiety and other related disorders: 4.6% – Mood disorders: 3.8% – Sleep disorder: 3.4% – Emotional state symptoms and signs: 0.8% – Suicidal ideation: 0.2%
Parra et al., 2020	COVID-19 patients with new-onset psychotic symptoms	Spain	10	6/4	54.1 (10.67)	–	Prevalence of symptoms: – Delusions: 100% – Orientation/attention disturbances: 60% – Auditory hallucinations: 40% – Visual hallucinations: 10%
Rogers et al., 2020	Review of 12 studies (including 7 preprints)	Global China: 10 studies Others: France, Japan	–	–	–	–	Evidence of neuropsychiatric manifestations: – Confusion: 5 studies – Anxiety and depression: 2 studies – Insomnia: 1 study
Romero-Sanchez et al., 2020	Hospitalized patients with COVID-19	Spain	841	473/368	66.42(14.96)	–	Prevalence of symptoms: – Insomnia: 13% – Anxiety: 8.1% – Depression: 5.2% – Psychosis: 1.3%
Varatharaj et al., 2020	CoroNerve Platform COVID-19 hospitalized patients with neurological manifestations	UK	125	73/44 (*36 not reported*)	–	–	Prevalence of manifestations: – Psychosis: 8% – Other psychiatric disorders: 5.6%
Vindegaard and Benros, 2020	Review of 43 studies: – *2 studies* of COVID-19 patients – 41 studies of health care workers, general public and psychiatric patients without COVID-19	Global	–	–	–	–	Evidence of neuropsychiatric manifestations: – High prevalence of PTSD in COVID-19 patients – Prevalence of depression higher in COVID-19 patients than in individuals under quarantine – Worsening of psychiatric symptoms in patients with pre-existing psychiatric disorders – Increased psychiatric symptoms in health care workers – Lower psychological well-being and higher scores in anxiety/depression scales after pandemic
Yuan et al., 2020	Cured COVID-19 patients in quarantine after discharge from hospital Subsample of Cai et al., 2020	China	96 (42 self-reported depression, 54 control group)	Depression group: 20/22 Control group: 27/27	Depression group: 49.6 (13.2) Control group: 45.2 (13.2)	Online questionnaire consisting of SDS scale:	Increased immune response (white blood cells count, neutrophil count and neutrophil-to-lymphocyte ratio) in the depression group in comparison to the control group
Zhang et al., 2020	COVID-19 patients in comparison to individuals under quarantine and general public	China	57 COVID-19 patients 50 individuals under quarantine 98 general public	29/28	46.9 (15.37)	App-based questionnaire – 9-item Patient Health Questionnaire (PHQ-9) – 7-item Generalized Anxiety Disorder Scale (GAD-7)	Percentage of subjects with COVID-19 who met the cut-off value of the scales: – Depression: 29.2% – Anxiety: 20.8% – Depression and anxiety: 21.1% Prevalence of depression was higher in COVID-19 patients than individuals under quarantine

*ARDS, Acute respiratory distress syndrome; ICU, Intensive care unit; ICD-10, 10th Revision of the International Statistical Classification of Diseases and Related Health Problems; PTSD, Post-traumatic stress disorder; UK, United Kingdom; US, United States.*

A surveillance study in the United Kingdom (UK) showed that 39 cases of a cohort of 125 COVID-19 hospitalized patients with neurological manifestations presented with altered mental status, with encephalopathy in 16 and neuropsychiatric syndromes in 23 of them, mostly new-onset psychosis (*n* = 10) or other related psychiatric disorders (*n* = 7) (Varatharaj et al., 2020). In line with this evidence, in a retrospective descriptive study from a hospital in Madrid, Spain, 10 patients with a lab-confirmed diagnosis of COVID-19 and new-onset psychotic symptoms were identified among 10,000 patients with symptoms compatible with COVID-19 assessed between March and April 2020 in the emergency department (Parra et al., 2020). They had a mean age of 54.1 years, and psychiatric symptoms, mainly delusion, orientation/attention disturbances and auditory hallucination (in 10, 6, and 4 cases, respectively), appeared primarily after the first typical COVID-19 symptoms and were resolved in less than 2 weeks (Parra et al., 2020). These episodes were considered atypical since patients had no familiar history of psychiatric disorders, no substance use disorders, had an atypical age of onset, and presented a fast recovery (Parra et al., 2020). Authors suggested that these atypical psychotic episodes may be explained by systemic inflammatory responses, based on analytical and complementary tests findings, or by side effects associated with COVID-19 treatment (Parra et al., 2020).

Critically ill patients with COVID-19 who require Intensive Care Unit (ICU) admission are also at most at risk of developing delirium, which is further exacerbated by the frequent need for high doses of sedation, elderly age and the presence of multiple comorbidities (Cipriani et al., 2020). In an observational study in France, 40 out of 58 (69%) COVID-19 patients attended in the ICU showed agitation and 26 of them confusional state (Helms et al., 2020). Brain imaging found bilateral frontotemporal hypoperfusion in eleven patients and larger leptomeningeal spaces in eight of them (Helms et al., 2020).

There is also evidence of prevalent depressive symptoms in those already recovered from COVID-19 (Cai et al., 2020; Yuan et al., 2020; Zhang et al., 2020). A study of 126 COVID-19 survivors in convalescence from Shenzhen, China, showed that self-reported anxiety and depression were common after discharge from hospital (Cai et al., 2020) and moreover, depressive symptoms were associated with immune systemic suppression, based on increased white blood cells and inflammatory factors measures (Yuan et al., 2020).

Despite this preliminary evidence, most of the findings came from self-reported scales, without clinical diagnostic assessments, and further examinations and follow-ups are needed to determine not only if these symptoms are related to the infection itself, secondary immune responses, side effects of treatments or psychologic stressors, but also if they improve, remain or worsen over time.

## Cognitive Manifestations of COVID-19

There are very few studies reporting cognitive symptoms related to COVID-19 (see Table 2). Data from 431,051 participants of the United Kingdom Biobank prospective study show that several psychosocial factors were associated with the risk of being hospitalized due to COVID-19, but after controlling for other relevant variables (sociodemographic, socioeconomic, psychological, lifestyle factors, and medical comorbidities), the only significant factor associated with the risk of the infection was a lower cognitive function (Batty et al., 2020). However, the causality and mechanisms involved in such association remain to be elucidated. In a retrospective study carried out in Chicago, United States, of 50 hospitalized patients with COVID-19 who were admitted to a neurology unit or presented neurological symptoms, 24% of them had short-term memory loss (Pinna et al., 2020). A surveillance study in the United Kingdom showed that 6 cases of a cohort of 125 COVID-19 hospitalized patients with neurological manifestations presented a neurocognitive disorder (Varatharaj et al., 2020).

**TABLE 2:** Cognitive manifestations of COVID-19.

Study	Type of COVID-19 sample	Country	N	Sex (M/F)	Age mean (SD)	Type of assessment	Results
Batty et al., 2020	UK Biobank data of COVID-19 hospitalized patients	UK	908 individuals with hospitalization due to COVID-19 430,143 no COVID-19	COVID-19 group: 506/402 No COVID-19 group: 193,820/236,323	COVID-19 group:57.27 (8.99) No COVID-19 group: 56.36 (8.10)	Computerized cognitive assessment at baseline (2006-2010)	Risk of COVID-19 hospitalization related to (after controlling for all covariates) cognitive function at baseline (verbal and numerical reasoning): Odds ratio 1.31
Chaumont et al., 2020	COVID-19 patients with ARDS and neurological manifestations admitted to an ICU	France	4	4/0	range 50-72	–	Prevalence of cognitive impairment: 100%
Helms et al., 2020	Patients admitted to an ICU with ARDS due to COVID-19	France	58	–	63 (median)	–	Prevalence of dysexecutive syndrome at discharge: (14/39) 36%
McLoughlin et al., 2020	COVID-19 hospitalized patients	UK	71 (16 no delirium, 31 delirium, 24 no assessment). 26 patients with a 4-week follow-up	51/20	61 (range 24-91)	Telephone assessment: – Telephone Instrument for Cognitive Status (TICS-m)	Cognitive performance at 4-week follow-up: Delirium group TICS-m mean score: 34.5/53 No delirium group TICS-m mean score: 41.5/53
Pinna et al., 2020	COVID-19 Hospitalized patients admitted to a neurology unit or with neurological symptoms	US	50	29/21	59.6 (14.3)	–	Prevalence of short-term memory loss: 24%
Varatharaj et al., 2020	CoroNerve Platform COVID-19 hospitalized patients with neurological manifestations	UK	125	73/44 (*36 not reported*)	–	–	Prevalence of neurocognitive disorder: 4.8%

*ARDS, Acute respiratory distress syndrome; ICU, Intensive care unit; UK, United Kingdom; US, United States.*

There is also preliminary evidence of cognitive impairment after hospital discharge. In this sense, in an observational study in France, over one third (15/45) of patients showed evidence of cognitive impairment at discharge from ICU, especially in the form of dysexecutive syndrome characterized by inattention, disorientation and poorly organized movements in response to commands (Helms et al., 2020). In a case series of 4 severe COVID-19 patients who required ICU admission, cognitive impairment, identified as memory deficit and frontal syndrome, was detected after discharged but remitted after 5 days of immunoglobulin therapy (Chaumont et al., 2020). Besides, in a sample of 71 COVID-19 hospitalized patients, those who were diagnosed with delirium during their hospitalization (42%) had lower cognitive scores on a telephone screening interview after 4 weeks of discharge, although the between-group comparison did not reach statistical significance (*p* = 0.06) (McLoughlin et al., 2020).

The lack of more precise information regarding cognitive symptoms in COVID-19 patients may be explained by the impact that the pandemic has had on healthcare systems and also, in severe cases, the difficulty to carry out a comprehensive neuropsychological assessment. However, this information will be of great value in order to identify risk factors related to the acute cognitive symptoms associated to the disease, both in people with or without pre-existing cognitive impairment, and to shed light on their underlying mechanisms. It will also be necessary to offer neuropsychological rehabilitation to those who need it. It is essential and urgent to minimize the potential negative effects on cognitive and psychosocial functioning and quality of life on survivors.

## Potential COVID-19 Cognitive and Neuropsychiatric Long-Term Complications

Long-term complications in those patients who survived the disease are currently unknown but are expected to appear in the next months and years, as it was seen in past pandemics caused by influenza or similar coronaviruses such as MERS-CoV and SARS-CoV (Rogers et al., 2020; Troyer et al., 2020) and also in survivors of critical illness who required ICU support (Desai et al., 2011; Herridge et al., 2016; Marra et al., 2018).

In a systematic review and meta-analysis carried out by Rogers et al. (2020) regarding the acute and post-illness neuropsychiatric manifestations of coronavirus infections, 72 studies were evaluated, including SARS-CoV (*n* = 47), MERS-CoV (*n* = 13) and the current SARS-CoV-2 (*n* = 12) and the hospitalized patients’ ages ranged from 12 to 68 years (Rogers et al., 2020). SARS-Cov and MERS-CoV were associated with prevalent neuropsychiatric symptoms both in the acute phase and after recovery (Rogers et al., 2020). Results from the meta-analysis carried out showed that after recovery, the estimated prevalence for PTSD was 32.2% (mean follow-up of 33.6 months) and prevalence of anxiety and depression disorders was 15% (mean follow-up of 11.6 and 22.6 months, respectively) (Rogers et al., 2020). Measures of health-related quality of life were significantly lower in patients compared to the control group and 76.9% (range: 66–93%) had returned to work after a mean of 3 years of follow-up (range: 1–144 months) (Rogers et al., 2020). As noted by the authors, most of the peer-reviewed studies included in the systematic review were considered either of low (32/65) or medium (30/65) quality due to limited assessment of previous psychiatric symptoms in the patients and lack of control group in many of them in order to distinguish symptoms related to the viral infection from the psychiatric impact these epidemics may had in the general population (Rogers et al., 2020). However, results from one of the longitudinal studies included in this review, with a sample of 181 individuals who had been infected by SARS-CoV-1, indicated that while only 3.3% of patients had a previous history of psychiatric disorder before the viral infection, after a mean follow-up of 3.4 years, 42.5% met clinical criteria of at least one psychiatric illness, being PTSD the most prevalent disorder (54.5%) followed by depression (39%) (Lam et al., 2009). Likewise, 70.8% of confirmed MERS-CoV cases developed psychiatric symptoms and 40% of them met clinical psychiatric diagnosis during hospital admission, while none of the suspected cases who were quarantined but tested negative for the virus showed any psychiatric symptoms (Kim et al., 2018). This evidence therefore suggests the possible role of coronavirus infections in inducing brain changes related to these psychiatric symptoms.

Moreover, ICU admission and invasive treatments such as ventilation and sedation following acute respiratory distress syndrome (ARDS) are risk factors for cognitive decline (Sasannejad et al., 2019). In the current pandemic caused by SARS-CoV-2, a retrospective case series study of 1591 COVID-19 patients admitted to ICU in Italy found that 88% required mechanical ventilation (Grasselli et al., 2020) and in a smaller study from an ICU in Washington, United States, 71% of 21 critically ill patients developed ARDS and required mechanical ventilation (Arentz et al., 2020). Data of long-term outcomes in adults requiring mechanical ventilation showed cognitive impairment in attention, memory, verbal fluency, processing speed or executive function in 78 and 47% of the patients after 1 and 2 years of discharge, respectively (Hopkins et al., 1999, Hopkins et al., 2005). A total of 15 patients in this study also underwent brain imaging and, in comparison to an age and sex-matched control group, they had significantly larger ventricular and temporal horn volumes (Hopkins et al., 2006). Even after 5 years of follow-up, 20% of survivors of ARDS showed cognitive sequelae in a wide variety of cognitive domains (Herridge et al., 2016).

## Mechanisms Involved in Cognitive and Neuropsychiatric Manifestations of COVID-19

**FIGURE 1:**
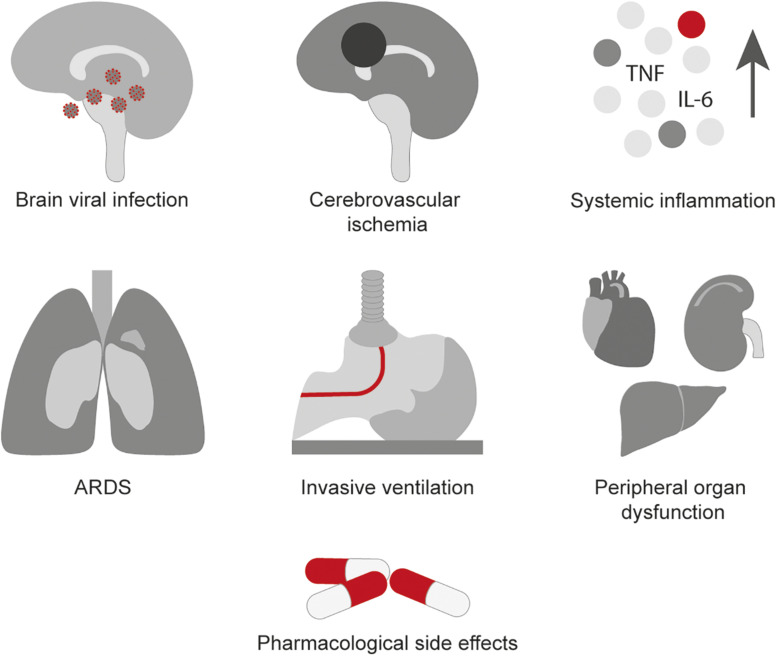
Possible mechanisms involved in the neurological manifestations of COVID-19.

The underlying causes for these symptoms related to COVID-19 and the mechanisms involved in potential long-lasting impairments are currently not fully understood but are probably multifactorial. These factors include direct viral infection of the nervous system, the systemic inflammatory response to the virus, cerebrovascular ischemia due to endothelial dysfunction or severe coagulopathy, the ARDS presented in severe cases, the use of invasive ventilation and sedation along with side effects of drugs used to treat COVID-19, and peripheral organ dysfunction (see Figure 1; Sasannejad et al., 2019; Heneka et al., 2020; Ogier et al., 2020).

SARS-CoV-2, similarly to other coronaviruses, shows certain neurotropism. Two potential methods for coronaviruses intracranial spread have been hypothesized: direct hematogenous attack and retrograde ascent via peripheral nerve fibers of the upper respiratory tract (Zubair et al., 2020). SARS-CoV-2 uses the SPIKE proteins located on its surface to bind the angiotensin-converting enzyme 2 (ACE2) receptor on mammalian host cells (Hoffmann et al., 2020). The ACE2 receptor has been found to be expressed widespread in neurons and glial cells (Xia and Lazartigues, 2008). There is evidence of neuronal death after brain infection by SARS-CoV-1 via the olfactory bulb in animal models (Netland et al., 2008).

To date there is little evidence of direct brain infection related to COVID-19. Results from reverse transcription polymerase chain reaction (RT-PCR) assays of cerebrospinal fluid (CSF) samples carried out in a few COVID-19 cases with neurological manifestations have been negative for SARS-CoV-2 (Chaumont et al., 2020; Helms et al., 2020).

Sparse neuropathological data of COVID-19 cases are available and mostly show hypoxic changes and demyelinating lesions (Coolen et al., 2020; Reichard et al., 2020; Solomon et al., 2020). An autopsy series from Germany detected SARS-CoV-2 viral load in brain along kidneys, liver, heart and blood, although in lower levels that in the respiratory system (Puelles et al., 2020). Further studies are needed to determine whether these lesions are due to SARS-CoV-2 infection or caused by illness-related secondary conditions. A recent systematic review that included 26 neuroimaging studies, most of them case series of COVID-19 patients who underwent brain imaging examination due to neurological symptoms, found that 34% (124/361) of the cases presented brain lesions probably attributable to COVID-19 and among them, the most common finding was diffuse subcortical and deep white matter abnormalities. Other common findings, although less prevalent, were microhemorrhages, hemorrhages, and infarcts (Rita Egbert et al., 2020).

Even in the absence of direct brain infection, severe systemic infection may be also involved by precipitating neuroinflammatory responses that may promote subsequent brain tissue damage (Frank-Cannon et al., 2009; Dantzer, 2018; Rea et al., 2018). In severe cases, the virus may trigger an exacerbated and dysregulated host response called “cytokine storm” that involves increased levels of pro-inflammatory cytokines such as necrosis tumoral factor (TNF) and interleukin-6 (IL-6), among others. If this response persists over time it creates a state of systemic inflammation, resulting in disruption of blood-brain barrier and neural and glial cells damage that can be involved in long-term sequelae in survivors. Current evidence related to SARS-CoV-2 shows that usually the most severely affected patients presented increased levels of pro-inflammatory cytokines (Chen et al., 2020; Huang et al., 2020; Yang et al., 2020).

Chronic systemic inflammation has been also studied as one of the underlying pathogenic mechanisms involved in neurodegenerative disease such as Alzheimer’s disease (AD) (Akiyama et al., 2000). In a sample of 12,336 participants with a mean age of 56.8 years, systemic inflammation was studied using a composite score of blood biomarkers, and results indicated a significant association between baseline inflammation and accelerated cognitive decline after a follow-up of 20 years (Walker et al., 2019). The inflammation related to viral infection significantly worsens tau-related pathology and results in impairment of spatial memory (Sy et al., 2011). The hippocampus, region involved in memory formation, is an especially vulnerable area to respiratory viral infections, as shown in animal models (Jacomy et al., 2006). Short-term deterioration in hippocampus-dependent learning and reduced long-term potentiation associated with impairment in spatial memory were observed in mice infected with the influenza virus (Hosseini et al., 2018). Also, in the presence of pro-inflammatory cytokines the microglial cells lose its capacity to phagocyte β-amyloid that can be related to the accumulation of amyloid plaques, one of the hallmarks of AD (Koenigsknecht-Talboo and Landreth, 2005).

The APOE-ε4 gene allele, the strongest genetic risk factor for AD, has been found to be linked to increased risk of infection and mortality due to COVID-19, although the biological mechanisms involved in this association remain to be known (Kuo et al., 2020). However, ACE2 expression has been reported to be reduced in mid-frontal brain tissue in AD patients, particularly in those carrying an APOE ε4 allele, and this reduction was negatively correlated with Aβ and phosphorylated tau pathology (Kehoe et al., 2016). It is also worth noting that in a study of 46 AD patients and 44 non-AD elderly individuals, the APOE-ε4 allele was overrepresented in AD patients positive for herpes simplex virus type 1 (HSV1) in the brain in comparison to those negative for the virus and even to non-AD patients positive for HSV1, with an OR of 16.8 (Itzhaki et al., 1997). Authors argued that APOE-ε4 could promote vulnerability to viral infection and neurodegeneration, participating in the extent of cells infected by the virus or in the extent of repair after HSV1-induced damage. Thus, viral infection may be an aggravating factor for neurodegeneration in individuals with susceptible genetic variants.

Despite this preliminary evidence related to COVID-19, further studies are needed in order to ascertain the underlying causes for these symptoms and the mechanisms involved in potential long-lasting impairments, and furthermore, whether this coronavirus might precipitate or exacerbate neurodegenerative diseases.

## COVID-19 Manifestations in Patients With Dementia

Elderly people with chronic diseases such as dementia develop more serious and often lethal forms of COVID-19 (Bianchetti et al., 2020; Hwang et al., 2020; Miyashita et al., 2020). People with dementia are likely unable to follow the recommendations from public health systems to reduce the transmission of the virus (such as physical distancing, frequent hand washing, and use of facial masks) (Suzuki et al., 2020), exposing them to a higher chance of infection. A review of 627 patients admitted to an acute medical ward with COVID-19 pneumonia in Italy revealed that those with dementia (*n* = 82, 13.1%) had a significantly higher mortality rate compared to those cognitively unimpaired (62.2% vs. 26.2%, *p* < 0.001) (Bianchetti et al., 2020). The diagnosis of dementia was independently associated with a higher mortality, with an odds ratio of 1.84 (Bianchetti et al., 2020). In the largest population-based study carried out to date and including 17,278,392 probable COVID-19 cases from England, previous history of stroke or dementia was associated with increased risk of death after 90 days, with an adjusted hazard ratio of 2.16 (Williamson et al., 2020). Similarly, in a nursing facility located in Washington, United States (US), among 101 residents who were positive for COVID-19, the case fatality rate (CFR, proportion of deaths from the total number of people diagnosed) associated was dramatically high (33.7%) (McMichael et al., 2020). Something to take into account is that given the rapid evolution of COVID-19 cases that have required medical attention, there is concern that older people with dementia may have been largely excluded from resources such as hospital admission and/or access to ICUs in favor of younger individuals or those with less comorbidities. This could partly explain the high mortality rates in this population and particularly, in long-term care and nursing home facilities (Cipriani and Fiorino, 2020).

Moreover, patients with dementia often present atypical symptoms, such as altered mental status (including confusion, agitation, disorientation, refusal of care, disorientation, and loss of appetite) as the initial COVID-19 manifestation, even without fever and cough (Isaia et al., 2020; Ward et al., 2020), along with worsening of baseline functional status (Bianchetti et al., 2020). Thus, waiting for typical respiratory symptoms to appear would delay appropriate diagnosis and treatment. Indeed, the Alzheimer’s Association advises caregivers to be alert to the presence of confusion as it is might be the first symptom of a possible COVID-19 infection in individuals with dementia (Alzheimer’s Association, 2020).

When treating COVID-19 in patients suffering from AD, drug interactions should be taken into account (Balli et al., 2020). Cholinesterase inhibitors (ChEIs) levels may increase during chloroquine (CQ), hydroxychloroquine (HCQ) and lopinavir/ritonavir treatment, due to effects on cytochrome P450. Cardiac adverse events can be related to both Azithromycin, CQ, HCQ, and ChEIs, so frequent electrocardiography monitoring is advised. Memantine has a low risk of pharmacokinetic interactions and may be a safer alternative when using drugs to treat COVID-19 in AD patients. Antipsychotics and antidepressants, used frequently in dementia, have potential interactions with Azithromycin, CQ, HCQ and lopinavir/ritonavir. Lastly, Tocilizumab, ribavirin and favipiravir show no potential interactions with AD treatments (Balli et al., 2020; University of Liverpool, 2020).

## COVID-19 Indirect Effects in Patients With Dementia

The COVID-19 pandemic further exacerbates the vulnerability of elderly patients with cognitive impairment, especially those who depend on family or caregivers for their daily care. This is due to the increased morbidity and mortality caused by the infection but also to the indirect effects of the pandemic on the healthcare system that they depend on (Brown et al., 2020). Medical resources have been diverted away from patients with chronic conditions, such as dementia, to attend COVID-19 cases. People with dementia are at risk of discontinuing their treatment during lockdown, especially those who depend on external help for reminders or assistance (Brown et al., 2020).

Most COVID-19-related deaths have occurred in long-term care facilities, where patients with dementia are a significant part of the residents and require close contact for assistance in their daily care (Blackman et al., 2020; Cordasco et al., 2020). Blackman et al. (2020) reported the rapid evolution of COVID-19 in a long-term care facility with 150 beds for individuals with dementia, where despite the preventive measures developed, within 3 weeks of the first confirmed COVID-19 case, 30 residents had died and more than 50 were confirmed cases or were having symptoms compatible for the disease. It is thus of crucial importance to equip these facilities with appropriate preventive measures and rapid detection capacity to avoid transmission and protect this already fragile population as much as possible.

The COVID-19 pandemic forced Memory Clinics worldwide to close their face-to-face consultations and non-pharmacological interventions for dementia, such as cognitive therapy, exercise and socialization, have been suddenly stopped during lockdown (Benaque et al., 2020; Brown et al., 2020). Many centers, though, took action and implemented telemedicine in order to continue assisting patients during the pandemic, both for their scheduled follow-up visits but also for urgent consultations (Benaque et al., 2020; Ousset and Vellas, 2020; Padala et al., 2020). This continued care during the pandemic has been especially important for those patients and caregivers in a more vulnerable situation. The transformation of medical care, though, posed a challenge for many patients, especially elderly individuals with cognitive, visual and/or hearing impairment, those living alone, and those from a low socio-economic status or living in rural areas with limited access to technology. From the clinical point of view, safety and legal concerns about privacy and data protection had to be addressed, and some tests from the neuropsychological batteries had to be adapted to be performed in a virtual way (Bilder et al., 2020). After this experience, it is likely that telemedicine will continue to have an important role in the evaluation of patients with cognitive impairment even after the COVID-19 pandemic is contained.

Community measures implemented to slow the spread of COVID-19 have forced to social distancing and self-isolation, and this may have contributed to generate feelings of loneliness and behavioral changes in patients with cognitive impairment (Canevelli et al., 2020). A significant proportion of patients with dementia reside in long-term care and nursing home facilities, which have been quarantined in many countries, prohibiting family members to visit residents or them to get outside. Factors such as solitariness and social isolation should not be underestimated, since they have been consistently found to be related to increased risk of medical health problems, including cardiovascular disease, depression or dementia (Holwerda et al., 2014; Courtin and Knapp, 2017), and higher mortality risk, even after controlling for relevant variables such as health status (Holt-Lunstad et al., 2015).

In this sense, there is evidence of cognitive, neuropsychiatric and functional worsening in this population during confinement periods implemented in several countries (see Table 3). A study from a cognitive disorders unit in Spain evaluating 40 patients with mild AD dementia and mild cognitive impairment (MCI) that attend a cognitive stimulating program reported that their neuropsychiatric symptoms significantly worsened after 5 weeks of lockdown (mainly apathy, anxiety, agitation and aberrant motor behavior) (Lara et al., 2020). Likewise, a report of 139 patients with dementia, MCI and subjective cognitive decline from a dementia center in Rome, showed worsening or onset of behavioral disturbances in 54.7% (mostly agitation/aggression, apathy, and depression) after 1 month of lockdown (Canevelli et al., 2020). In 7.2% of cases, these symptoms required adjustments or introduction of pharmacological treatments, mostly antipsychotics (Canevelli et al., 2020). Worsening of neuropsychiatric symptoms has been found particularly associated with significant lower general cognitive functioning before confinement in a sample of 38 patients with a clinical diagnosis of probable AD (Boutoleau-Bretonniere et al., 2020). In a sample of 93 older adults with MCI or mild dementia, those living alone reported significantly a decrease in their well-being, more anxiety and sleeping problems (Goodman-Casanova et al., 2020). Also, in a study with a smaller sample of 32 individuals with frontotemporal lobar dementia from a dementia care center in Tricase (Italy), caregivers were interviewed by telephone using a structured clinical assessment and reported that, compared to their last visit (mean of 6.78 months), 53% of patients showed significant worsening in cognitive function, particularly in memory, along with worsening in behavior and language function during COVID-19 confinement (Capozzo et al., 2020).

**TABLE 3:** Neuropsychiatric and cognitive manifestations in dementia patients during confinement.

Study	Country	Participants	Male/Female	Age mean (SD)	Assessment	Results
Lara et al., 2020	Spain	40 – Mild AD: 20 – MCI: 20	16/24	77.4 (5.25)	Phone interview after 5 weeks of home confinement: – NPI – EuroQol-5D	Significant worsening in neuropsychiatric symptoms after confinement (NPI score baseline: 33.75 vs. confinement: 39.05) No changes in quality of life (0.66 vs. 0.62)
Canevelli et al., 2020	Italy	139 – Dementia: 96 – MCI: 37 – SCD: 6	55/84	79	Telephone survey in patients or caregivers	Percentage of individuals with reported worsening: – Behavioral: 54.7% – Neuropsychiatric symptoms: 54.68% – Cognition: 31.65% – Functional decline 13.67% – Required adjustment/introduction of pharmacological treatments: 7.2%
Boutoleau-Bretonniere et al., 2020	France	38 individuals with probable AD	15/23	71.89 (8.24)	Caregivers phone interview: – NPI	Prevalence of neuropsychiatric worsening (mean duration of confinement: 27.37 days): 26.31% Worsening associated with lower general cognitive functioning before confinement (2-4 months before).
Goodman-Casanova et al., 2020	Spain	93 individuals with MCI or mild dementia	33/60	73.34 (6.07)	Telephone-based survey in patients or caregivers	Prevalence of mental health and well-being status reported: – Sleep quality maintained: 70% – Well: 61%* – Sad: 29%* – Anxious: 24%* – Sleep quality altered: 24%* – Worried: 22% – Bored: 14% – Afraid: 11% – Calm: 9%
Capozzo et al., 2020	Italy	32 individuals with frontotemporal lobar dementia	18/14	66.25 (9.76)	Telemedicine assessment Structured clinical questionnaire in caregivers	Prevalence of individuals with reported worsening: – Cognitive function: 53% – Behavior: 56% – Language: 47% – Sleep disturbances: 25%

AD, Alzheimer’s disease; EuroQol-5D, European Quality of Life-5 Dimensions; MCI, Mild cognitive impairment; NPI, Neuropsychiatric inventory; SCD, Subjective cognitive decline; *, Significant differences between those living alone (*n* = 24) vs. living with others.

Another concern is the effect that this pandemic could have on those individuals at the preclinical stage of dementia or those experiencing subtle cognitive changes. After 6 months with the healthcare systems worldwide on edge, referrals to Memory Clinics and timely diagnosis and interventions will probably be delayed. Prevention is the most important strategy in potentially slowing the progression of neurodegenerative disorders and given the increased risk of negative health outcomes in older people, it is important to examine and determine whether COVID-19 may trigger or aggravate neurodegenerative processes in this vulnerable group.

## COVID-19 Effects on Dementia Research and Clinical Trials

A recent survey among 267 researchers and clinicians specialized in dementia identified 9 priorities in research for older people, including management of COVID-19 and its complications and outcomes in this population, and the need to include older people in research studies to design safe and adequate interventions (Richardson et al., 2020). However, while clinical trials should be designed to enable inclusion of elderly individuals due to the increased morbidity and mortality caused by the infection in this population, a recent review reported that over a third of COVID-19-related clinical trials registered as of 16th March 2020 excluded participants over 75 years of age (Lithander et al., 2020).

The closure of Memory Clinics due to the lockdown measures imposed by governments worldwide has had a negative effect on dementia clinical research as well. Although some procedures could be still performed through telemedicine such as clinical evaluations and neuropsychological testing, this type of research usually requires the presence of patients to undergo blood draw, lumbar puncture or neuroimaging, among others.

Basic laboratories conducting experimental research in the Alzheimer’s field have also been affected by COVID-19, as the sudden lack of staff due to lockdown forced ongoing experiments to be stopped and transgenic animals to die, with the resulting loss of valuable data (Bostanciklioglu, 2020). Besides, research funding is currently being diverted into COVID-19 research, leaving dementia researchers with less funding opportunities to continue their projects. Also, until movement restriction measures among countries are lifted, international students and researchers will not be allowed to continue or start new positions in foreign countries.

The COVID-19 pandemic has compelled most ongoing clinical trials in the AD field to be put on hold for a few months, affecting participants’ recruitment, administration of medication infusions and follow-up visits (Weinberg et al., 2020). This has caused a significant disruption in the management of clinical trials, with the need of protocol amendments to deal with missing data, deviations, loss of participants and allowing the use of telemedicine. In the United States, the Food and Drug Administration (FDA) issued on April 2nd a document entitled “Guidance on Conduct of Clinical Trials of Medical Products during the COVID-19 Pandemic” (The Food and Drug Administration, 2020), encouraging sponsors to use their best judgment to decide the continuance of the clinical trial during the pandemic. Lastly, the clinical trials disruption has especially affected patients and their families in a personal manner, as they trusted pharmacological research to fight against this disease, faced great uncertainty and helplessness regarding their future, but in general were eager to continue their treatment once it was safe (Geerts and van der Graaf, 2020; Weinberg et al., 2020).

Something to be considered is the need for SARS-CoV-2 RT-PCR testing prior to the onset of treatment once participants are allowed to reinitiate their participation in clinical trials, as some novel drugs being tested against Alzheimer have immune-mediated mechanisms (Perez-Grijalba et al., 2019) and COVID-19 should to be ruled out for safety reasons.

Given the impact that this pandemic is having on elderly people with dementia and based on the evidence reviewed and existing to date, a summary of relevant considerations to take into account for this population is presented in Table 4.

**TABLE 4:** Summary of considerations for dementia management and research in relation to COVID-19.

Health and social care	Implement telemedicine to continue consultations and non-pharmacological interventions
Health and social care	Implement technology solutions to facilitate communication with family members during quarantine or confinement periods.
	Facilitate solutions to continue pharmacological treatment to those who depend on external help for reminders or assistance during quarantine and confinement periods
Health and social care	Continue the assessment of individuals with cognitive complaints to facilitate early diagnosis and interventions as prevention of neurodegenerative progression
Prevention	Equip long-term facilities with appropriate preventive measures and rapid detection capacity to avoid COVID-19 transmission
Prevention	Be aware of the onset of COVID-19 as atypical symptoms/worsening of baseline status
Treatment	Take into account pharmacological interactions when treating for COVID-19 in this population
Treatment	Perform follow-up evaluations to COVID-19 survivors and provide mental health support and cognitive rehabilitation when necessary
Research and clinical trials	Include older people in research studies to design safe and adequate interventions
Research and clinical trials	Continue research funding in dementia
Research and clinical trials	Continue dementia clinical trials, considering testing for SARS-CoV-2 prior to the onset of treatment.
Research and clinical trials	Perform follow-up evaluation to COVID-19 survivors to examine the presence of long-term neuropsychiatric and cognitive complications

## Conclusion

The current COVID-19 pandemic is having a significant impact on many health, economic and social aspects worldwide. Elderly population, and especially those with comorbidities such as dementia, is a vulnerable group at risk of contracting the disease and presenting more severe forms and worse outcomes, including mortality. In this regard, long-term care facilities have been specially hit by the pandemic, showing high rates of infection and mortality. Memory Clinics attending patients with dementia have been forced to cancel their face-to-face appointments, while research and clinical trials in the field of Alzheimer’s disease have also suffered the negative consequences of the pandemic.

At this point, it is of crucial relevance to perform follow-up evaluations to COVID-19 survivors using comprehensive cognitive and neuropsychiatric assessments along with brain imaging if appropriate, especially to those who have suffered severe forms of the disease with ICU-care level and neurological manifestations during the acute phase. It is necessary to rule out long-term sequelae and provide mental health support and cognitive rehabilitation to minimize the potential negative effects on psychosocial functioning and quality of life of survivors. Given the increased risk of negative health outcomes in older individuals, it is important to examine and determine whether COVID-19 may trigger or aggravate neurodegenerative processes in this vulnerable group, as an early diagnosis and intervention are the most important strategies to slow the progression of neurodegenerative disorders. Importantly, clinical trials and research studies related to COVID-19 should be designed to enable inclusion of elderly individuals.

## Author Contributions

SA-L and MM equally contributed to the scientific literature review and wrote the manuscript. SA-L designed the figure. AR and MB have supervised the study and have critically revised and approved the final version of the manuscript. All authors contributed to the article and approved the submitted version.

## Conflict of Interest

The authors declare that the research was conducted in the absence of any commercial or financial relationships that could be construed as a potential conflict of interest.

## References

[B1] Akiyama H. Barger S. Barnum S. Bradt B. Bauer J. Cole G. M. (2000). Inflammation and Alzheimer’s disease. *Neurobiol. Aging* 21 383–421. 10858586 10.1016/s0197-4580(00)00124-x PMC3887148

[B2] Alzheimer’s Association (2020). *Coronavirus (COVID-19): Tips for Dementia Caregivers.* Available online at: https://www.alz.org/help-support/caregiving/coronavirus-(covid-19)-tips-for-dementia-care (accessed July 24, 2020).

[B3] Arentz M. Yim E. Klaff L. Lokhandwala S. Riedo F. X. Chong M. (2020). Characteristics and outcomes of 21 critically ill patients with COVID-19 in Washington state. *JAMA* 323 1612–1614. 10.1001/jama.2020.4326 32191259 PMC7082763

[B4] Balli N. Kara E. Demirkan K. (2020). The another side of COVID-19 in Alzheimer’s disease patients: drug-drug interactions. *Int. J. Clin. Pract.* 74:e13596. 10.1111/ijcp.13596 PMC7361245 32593196

[B5] Batty G. D. Deary I. J. Luciano M. Altschul D. M. Kivimaki M. Gale C. R. (2020). Psychosocial factors and hospitalisations for COVID-19: prospective cohort study based on a community sample. *Brain Behav. Immun.* 10.1016/j.bbi.2020.06.021 [Epub ahead of print]. 32561221 PMC7297693

[B6] Benaque A. Gurruchaga M. J. Abdelnour C. Hernandez I. Canabate P. Alegret M. (2020). Dementia care in times of COVID-19: experience at Fundacio ACE in Barcelona. Spain. *J. Alzheimers Dis.* 76 33–40. 10.3233/jad-200547 32538856 PMC7369075

[B7] Bianchetti A. Rozzini R. Guerini F. Boffelli S. Ranieri P. Minelli G. (2020). Clinical presentation of COVID19 in Dementia patients. *J. Nutr. Health Aging* 24 560–562. 10.1007/s12603-020-1389-1 32510106 PMC7227170

[B8] Bilder R. M. Postal K. S. Barisa M. Aase D. M. Cullum C. M. Gillaspy S. R. (2020). InterOrganizational practice committee recommendations/guidance for teleneuropsychology (TeleNP) in response to the COVID-19 pandemic. *Clin. Neuropsychol.* 10.1080/13854046.2020.1767214 [Epub ahead of print]. 32673163 PMC7767580

[B9] Blackman C. Farber S. Feifer R. A. Mor V. White E. M. (2020). An illustration of SARS-CoV-2 dissemination within a skilled nursing facility using heat maps. *J. Am. Geriatr. Soc.* 10.1111/jgs.16642 [Epub ahead of print]. 32533847 PMC7323317

[B10] Borges Do Nascimento I. J. Cacic N. Abdulazeem H. M. Von Groote T. C. Jayarajah U. (2020). Novel Coronavirus infection (COVID-19) in humans: a scoping review and meta-analysis. *J. Clin. Med.* 9:941. 10.3390/jcm9040941 PMC7230636 32235486

[B11] Bostanciklioglu M. (2020). Severe acute respiratory syndrome coronavirus 2 is penetrating to dementia research. *Curr. Neurovasc. Res.* 10.2174/1567202617666200522220509 [Epub ahead of print]. 32442082

[B12] Boutoleau-Bretonniere C. Pouclet-Courtemanche H. Gillet A. Bernard A. Deruet A. L. Gouraud I. (2020). The effects of confinement on neuropsychiatric symptoms in Alzheimer’s disease during the COVID-19 crisis. *J. Alzheimers Dis.* 76 41–47. 10.3233/jad-200604 32568211 PMC9988367

[B13] Brown E. E. Kumar S. Rajji T. K. Pollock B. G. Mulsant B. H. (2020). Anticipating and mitigating the impact of the COVID-19 pandemic on Alzheimer’s disease and related dementias. *Am. J. Geriatr. Psychiatry* 28 712–721. 10.1016/j.jagp.2020.04.010 32331845 PMC7165101

[B14] Cai X. Hu X. Otte E. I. Wang J. An Y. Li Z. (2020). Psychological distress and its correlates among COVID-19 survivors during early convalescence across age groups. *Am. J. Geriatr. Psychiatry* 28 1030–1039. 10.1016/j.jagp.2020.07.003 32753338 PMC7347493

[B15] Canevelli M. Valletta M. Toccaceli Blasi M. Remoli G. Sarti G. Nuti F. (2020). Facing dementia during the COVID-19 outbreak. *J. Am. Geriatr. Soc.* 68 1673–1676. 10.1111/jgs.16644 32516441 PMC7300919

[B16] Capozzo R. Zoccolella S. Frisullo M. E. Barone R. Dell’abate M. T. Barulli M. R. (2020). Telemedicine for delivery of care in frontotemporal lobar degeneration during COVID-19 pandemic: results from Southern Italy. *J. Alzheimers Dis.* 76 481–489. 32651328 10.3233/JAD-200589

[B17] Centers for Disease Control and Prevention (2020). *COVIDView: A Weekly Surveillance Summary of US COVID-19 activity. Key updates for week, 29.* Available online at: https://www.cdc.gov/coronavirus/2019-ncov/covid-data/pdf/covidview-07-24-2020.pdf (accessed July 28, 2020).

[B18] Chaumont H. San-Galli A. Martino F. Couratier C. Joguet G. Carles M. (2020). Mixed central and peripheral nervous system disorders in severe SARS-CoV-2 infection. *J. Neurol.* 10.1007/s00415-020-09986-y [Epub ahead of print]. 32533322 PMC7292244

[B19] Chen G. Wu D. Guo W. Cao Y. Huang D. Wang H. (2020). Clinical and immunological features of severe and moderate coronavirus disease 2019. *J. Clin. Invest.* 130 2620–2629. 10.1172/jci137244 32217835 PMC7190990

[B20] Cipriani G. Danti S. Nuti A. Carlesi C. Lucetti C. Di Fiorino M. (2020). A complication of coronavirus disease 2019: delirium. *Acta Neurol Belg.* 120 927–932. 10.1007/s13760-020-01401-7 32524537 PMC7286634

[B21] Cipriani G. Fiorino M. D. (2020). Access to care for dementia patients suffering from COVID-19. *Am. J. Geriatr. Psychiatry* 28 796–797. 10.1016/j.jagp.2020.04.009 32327300 PMC7162751

[B22] Coolen T. Lolli V. Sadeghi N. Rovai A. Trotta N. Taccone F. S. (2020). Early postmortem brain MRI findings in COVID-19 non-survivors. *Neurology* 95 e2016–e2027. 10.1212/WNL.0000000000010116 32546654

[B23] Cordasco F. Scalise C. Sacco M. A. Bonetta C. F. Zibetti A. Cacciatore G. (2020). The silent deaths of the elderly in long-term care facilities during the Covid-19 pandemic: the role of forensic pathology. *Med. Leg. J.* 88 66–68. 10.1177/0025817220930552 32507030

[B24] Courtin E. Knapp M. (2017). Social isolation, loneliness and health in old age: a scoping review. *Health Soc. Care Community* 25 799–812. 10.1111/hsc.12311 26712585

[B25] Dantzer R. (2018). Neuroimmune interactions: from the brain to the immune system and vice versa. *Physiol. Rev.* 98 477–504. 10.1152/physrev.00039.2016 29351513 PMC5866360

[B26] Desai S. V. Law T. J. Needham D. M. (2011). Long-term complications of critical care. *Crit. Care Med.* 39 371–379. 10.1097/ccm.0b013e3181fd66e5 20959786

[B27] Dinakaran D. Manjunatha N. Naveen Kumar C. Suresh B. M. (2020). Neuropsychiatric aspects of COVID-19 pandemic: a selective review. *Asian J. Psychiatr.* 53:102188. 10.1016/j.ajp.2020.102188 32512530 PMC7261092

[B28] Ellul M. A. Benjamin L. Singh B. Lant S. Michael B. D. Easton A. (2020). Neurological associations of COVID-19. *Lancet Neurol.* 19 767–783. 10.1016/S1474-4422(20)30221-0 32622375 PMC7332267

[B29] Frank-Cannon T. C. Alto L. T. Mcalpine F. E. Tansey M. G. (2009). Does neuroinflammation fan the flame in neurodegenerative diseases? *Mol. Neurodegener.* 4:47. 10.1186/1750-1326-4-47 19917131 PMC2784760

[B30] Geerts H. van der Graaf P. H. (2020). Salvaging CNS Clinical Trials Halted Due to COVID-19. *CPT Pharmacometrics Syst. Pharmacol.* 9 367–370. 10.1002/psp4.12535 32468710 PMC7283764

[B31] Goodman-Casanova J. M. Dura-Perez E. Guzman-Parra J. Cuesta-Vargas A. Mayoral-Cleries F. (2020). Telehealth home support during COVID-19 confinement for community-dwelling older adults with mild cognitive impairment or mild dementia: survey study. *J. Med. Internet Res.* 22:e19434. 10.2196/19434 32401215 PMC7247465

[B32] Goujon A. Natale F. Ghio D. Conte A. Dijkstra L. (2020). *Age, Gender, and Territory of COVID-19 Infections and Fatalities, EUR 30237 EN.* Luxembourg: Publications Office of the European Union.

[B33] Grasselli G. Zangrillo A. Zanella A. Antonelli M. Cabrini L. Castelli A. (2020). Baseline characteristics and outcomes of 1591 patients infected with SARS-CoV-2 admitted to ICUs of the Lombardy Region, Italy. *JAMA* 323 1574–1581. 10.1001/jama.2020.5394 32250385 PMC7136855

[B34] Helms J. Kremer S. Merdji H. Clere-Jehl R. Schenck M. Kummerlen C. (2020). Neurologic features in severe SARS-CoV-2 infection. *N. Engl. J. Med.* 382 2268–2270. 10.1056/nejmc2008597 32294339 PMC7179967

[B35] Heneka M. T. Golenbock D. Latz E. Morgan D. Brown R. (2020). Immediate and long-term consequences of COVID-19 infections for the development of neurological disease. *Alzheimers Res. Ther.* 12:69. 10.1186/s13195-020-00640-3 PMC7271826 32498691

[B36] Herridge M. S. Moss M. Hough C. L. Hopkins R. O. Rice T. W. Bienvenu O. J. (2016). Recovery and outcomes after the acute respiratory distress syndrome (ARDS) in patients and their family caregivers. *Intensive Care Med.* 42 725–738. 10.1007/s00134-016-4321-8 27025938

[B37] Hoffmann M. Kleine-Weber H. Schroeder S. Kruger N. Herrler T. Erichsen S. (2020). SARS-CoV-2 cell entry depends on ACE2 and TMPRSS2 and is blocked by a clinically proven protease inhibitor. *Cell* 181 271–280.e8. 32142651 10.1016/j.cell.2020.02.052 PMC7102627

[B38] Holt-Lunstad J. Smith T. B. Baker M. Harris T. Stephenson D. (2015). Loneliness and social isolation as risk factors for mortality: a meta-analytic review. *Perspect. Psychol. Sci.* 10 227–237. 10.1177/1745691614568352 25910392

[B39] Holwerda T. J. Deeg D. J. Beekman A. T. Van Tilburg T. G. Stek M. L. Jonker C. (2014). Feelings of loneliness, but not social isolation, predict dementia onset: results from the Amsterdam Study of the Elderly (AMSTEL). *J. Neurol. Neurosurg. Psychiatry* 85 135–142. 10.1136/jnnp-2012-302755 23232034

[B40] Hopkins R. O. Gale S. D. Weaver L. K. (2006). Brain atrophy and cognitive impairment in survivors of Acute Respiratory Distress Syndrome. *Brain Inj.* 20 263–271. 10.1080/02699050500488199 16537268

[B41] Hopkins R. O. Weaver L. K. Collingridge D. Parkinson R. B. Chan K. J. Orme J. F. Jr. (2005). Two-year cognitive, emotional, and quality-of-life outcomes in acute respiratory distress syndrome. *Am. J. Respir. Crit. Care Med.* 171 340–347. 10.1164/rccm.200406-763oc 15542793

[B42] Hopkins R. O. Weaver L. K. Pope D. Orme J. F. Bigler E. D. Larson L. V. (1999). Neuropsychological sequelae and impaired health status in survivors of severe acute respiratory distress syndrome. *Am. J. Respir. Crit. Care Med.* 160 50–56. 10.1164/ajrccm.160.1.9708059 10390379

[B43] Hosseini S. Wilk E. Michaelsen-Preusse K. Gerhauser I. Baumgartner W. Geffers R. (2018). Long-term neuroinflammation induced by Influenza A virus infection and the impact on hippocampal neuron morphology and function. *J. Neurosci.* 38 3060–3080. 10.1523/jneurosci.1740-17.2018 29487124 PMC6596076

[B44] Huang C. Wang Y. Li X. Ren L. Zhao J. Hu Y. (2020). Clinical features of patients infected with 2019 novel coronavirus in Wuhan, China. *Lancet* 395 497–506. 31986264 10.1016/S0140-6736(20)30183-5 PMC7159299

[B45] Hwang J. M. Kim J. H. Park J. S. Chang M. C. Park D. (2020). Neurological diseases as mortality predictive factors for patients with COVID-19: a retrospective cohort study. *Neurol. Sci.* 41 2317–2324. 10.1007/s10072-020-04541-z 32643133 PMC7342552

[B46] Isaia G. Marinello R. Tibaldi V. Tamone C. Bo M. (2020). Atypical presentation of Covid-19 in an older adult with severe Alzheimer disease. *Am. J. Geriatr. Psychiatry* 28 790–791. 10.1016/j.jagp.2020.04.018 32381283 PMC7175908

[B47] Itzhaki R. F. Lin W. R. Shang D. Wilcock G. K. Faragher B. Jamieson G. A. (1997). Herpes simplex virus type 1 in brain and risk of Alzheimer’s disease. *Lancet* 349 241–244. 10.1016/s0140-6736(96)10149-5 9014911

[B48] Jacomy H. Fragoso G. Almazan G. Mushynski W. E. Talbot P. J. (2006). Human coronavirus OC43 infection induces chronic encephalitis leading to disabilities in BALB/C mice. *Virology* 349 335–346. 10.1016/j.virol.2006.01.049 16527322 PMC7111850

[B49] Kehoe P. G. Wong S. Al Mulhim N. Palmer L. E. Miners J. S. (2016). Angiotensin-converting enzyme 2 is reduced in Alzheimer’s disease in association with increasing amyloid-beta and tau pathology. *Alzheimers Res. Ther.* 8:50. 10.1186/s13195-016-0217-7 PMC5123239 27884212

[B50] Kim H. C. Yoo S. Y. Lee B. H. Lee S. H. Shin H. S. (2018). Psychiatric findings in suspected and confirmed middle east respiratory syndrome patients quarantined in hospital: a retrospective chart analysis. *Psychiatry Investig.* 15 355–360. 10.30773/pi.2017.10.25.1 29593206 PMC5912494

[B51] Koenigsknecht-Talboo J. Landreth G. E. (2005). Microglial phagocytosis induced by fibrillar beta-amyloid and IgGs are differentially regulated by proinflammatory cytokines. *J. Neurosci.* 25 8240–8249. 10.1523/jneurosci.1808-05.2005 16148231 PMC6725530

[B52] Kuo C. L. Pilling L. C. Atkins J. L. Masoli J. A. H. Delgado J. Kuchel G. A. (2020). ApoE e4e4 genotype and mortality with COVID-19 in UK Biobank. *J. Gerontol. A Biol. Sci. Med. Sci.* 75 1801–1803. 10.1093/gerona/glaa169 32623451 PMC7337688

[B53] Lam M. H. Wing Y. K. Yu M. W. Leung C. M. Ma R. C. Kong A. P. (2009). Mental morbidities and chronic fatigue in severe acute respiratory syndrome survivors: long-term follow-up. *Arch. Intern. Med.* 169 2142–2147. 10.1001/archinternmed.2009.384 20008700

[B54] Lara B. Carnes A. Dakterzada F. Benitez I. Pinol-Ripoll G. (2020). Neuropsychiatric symptoms and quality of life in Spanish patients with Alzheimer’s disease during the COVID-19 lockdown. *Eur. J. Neurol.* 10.1111/ene.14339 [Epub ahead of print]. 32449791 PMC7283827

[B55] Lechien J. R. Chiesa-Estomba C. M. Place S. Van Laethem Y. Cabaraux P. Mat Q. (2020). Clinical and epidemiological characteristics of 1420 European patients with mild-to-moderate coronavirus disease 2019. *J. Intern. Med.* 288 335–344. 10.1111/joim.13089 32352202 PMC7267446

[B56] Liguori C. Pierantozzi M. Spanetta M. Sarmati L. Cesta N. Iannetta M. (2020). Subjective neurological symptoms frequently occur in patients with SARS-CoV2 infection. *Brain Behav. Immun.* 88 11–16. 10.1016/j.bbi.2020.05.037 32416289 PMC7235586

[B57] Lithander F. E. Neumann S. Tenison E. Lloyd K. Welsh T. J. Rodrigues J. C. L. (2020). COVID-19 in older people: a rapid clinical review. *Age Ageing* 49 501–515. 32377677 10.1093/ageing/afaa093 PMC7239238

[B58] Lu L. Zhong W. Bian Z. Li Z. Zhang K. Liang B. (2020). A comparison of mortality-related risk factors of COVID-19, SARS, and MERS: a systematic review and meta-analysis. *J. Infect.* 81 e18–e25. 10.1016/j.jinf.2020.07.002 PMC7334925 32634459

[B59] Marra A. Pandharipande P. P. Girard T. D. Patel M. B. Hughes C. G. Jackson J. C. (2018). Co-occurrence of post-intensive care syndrome problems among 406 survivors of critical illness. *Crit. Care Med.* 46 1393–1401. 10.1097/ccm.0000000000003218 29787415 PMC6095801

[B60] Martin-Sanchez F. J. Del Toro E. Cardassay E. Valls Carbo A. Cuesta F. Vigara M. (2020). Clinical presentation and outcome across age categories among patients with COVID-19 admitted to a Spanish Emergency Department. *Eur. Geriatr. Med.* 10.1007/s41999-020-00359-2 [Epub ahead of print]. 32671732 PMC7363499

[B61] McLoughlin B. C. Miles A. Webb T. E. Knopp P. Eyres C. Fabbri A. (2020). Functional and cognitive outcomes after COVID-19 delirium. *Eur. Geriatr. Med.* 10.1007/s41999-020-00353-8 [Epub ahead of print]. 32666303 PMC7358317

[B62] McMichael T. M. Currie D. W. Clark S. Pogosjans S. Kay M. Schwartz N. G. (2020). Epidemiology of Covid-19 in a long-term care facility in King County, Washington. *N. Engl. J. Med.* 382 2005–2011. 32220208 10.1056/NEJMoa2005412 PMC7121761

[B63] Miyashita S. Yamada T. Mikami T. Miyashita H. Chopra N. Rizk D. (2020). Impact of dementia on clinical outcomes in elderly patients with coronavirus 2019 (COVID-19): an experience in New York. *Geriatr. Gerontol. Int.* 20 732–734. 10.1111/ggi.13942 32691924 PMC7404346

[B64] Nalleballe K. Reddy Onteddu S. Sharma R. Dandu V. Brown A. Jasti M. (2020). Spectrum of neuropsychiatric manifestations in COVID-19. *Brain Behav. Immun.* 88 71–74. 10.1016/j.bbi.2020.06.020 32561222 PMC7297688

[B65] Netland J. Meyerholz D. K. Moore S. Cassell M. Perlman S. (2008). Severe acute respiratory syndrome coronavirus infection causes neuronal death in the absence of encephalitis in mice transgenic for human ACE2. *J. Virol.* 82 7264–7275. 10.1128/jvi.00737-08 18495771 PMC2493326

[B66] Ogier M. Andeol G. Sagui E. Bo G. D. (2020). How to detect and track chronic neurologic sequelae of Covid-19? Use of auditory brainstem responses and neuroimaging for long-term patient follow-up. *Brain Behav. Immun. Health* 5:100081. 10.1016/j.bbih.2020.100081 32427134 PMC7227537

[B67] Ousset P. J. Vellas B. (2020). Viewpoint: impact of the Covid-19 outbreak on the clinical and research activities of memory clinics: an Alzheimer’s disease center facing the Covid-19 crisis. *J. Prev. Alzheimers Dis.* 7 197–198. 32463074 10.14283/jpad.2020.17 PMC7147199

[B68] Padala S. P. Jendro A. M. Orr L. C. (2020). Facetime to reduce behavioral problems in a nursing home resident with Alzheimer’s dementia during COVID-19. *Psychiatry Res.* 288:113028. 10.1016/j.psychres.2020.113028 32361337 PMC7177115

[B69] Parra A. Juanes A. Losada C. P. Alvarez-Sesmero S. Santana V. D. Marti I. (2020). Psychotic symptoms in COVID-19 patients. A retrospective descriptive study. *Psychiatry Res.* 291:113254. 10.1016/j.psychres.2020.113254 32603930 PMC7311337

[B70] Paterson R. W. Brown R. L. Benjamin L. Nortley R. Wiethoff S. Bharucha T. (2020). The emerging spectrum of COVID-19 neurology: clinical, radiological and laboratory findings. *Brain.* 10.1093/brain/awaa240 [Epub ahead of print]. 32637987 PMC7454352

[B71] Perez-GrijalbaV.RomeroJ.PesiniP.SarasaL.MonleonI.San-JoseI. (2019). Plasma Abeta42/40 ratio detects early stages of Alzheimer’s disease and correlates with CSF and neuroimaging biomarkers in the AB255 study. *J. Prev. Alzheimers Dis.* 6 34–41.3056908410.14283/jpad.2018.41

[B72] Pinna P. Grewal P. Hall J. P. Tavarez T. Dafer R. M. Garg R. (2020). Neurological manifestations and COVID-19: experiences from a tertiary care center at the Frontline. *J. Neurol. Sci.* 415:116969. 10.1016/j.jns.2020.116969 32570113 PMC7832569

[B73] Pinzon R. T. Wijaya V. O. Buana R. B. Al Jody A. Nunsio P. N. (2020). Neurologic characteristics in Coronavirus disease 2019 (COVID-19): a systematic review and meta-analysis. *Front. Neurol.* 11:565. 10.3389/fneur.2020.00565 32574250 PMC7273516

[B74] Puelles V. G. Lutgehetmann M. Lindenmeyer M. T. Sperhake J. P. Wong M. N. Allweiss L. (2020). Multiorgan and renal tropism of SARS-CoV-2. *N. Engl. J. Med.* 383 590–592. 10.1056/NEJMc2011400 32402155 PMC7240771

[B75] Rea I. M. Gibson D. S. Mcgilligan V. Mcnerlan S. E. Alexander H. D. Ross O. A. (2018). Age and age-related diseases: role of inflammation triggers and cytokines. *Front. Immunol.* 9:586. 10.3389/fimmu.2018.00586 29686666 PMC5900450

[B76] Reichard R. R. Kashani K. B. Boire N. A. Constantopoulos E. Guo Y. Lucchinetti C. F. (2020). Neuropathology of COVID-19: a spectrum of vascular and acute disseminated encephalomyelitis (ADEM)-like pathology. *Acta Neuropathol.* 140 1–6. 10.1007/s00401-020-02166-2 32449057 PMC7245994

[B77] Richardson S. J. Carroll C. B. Close J. Gordon A. L. O’brien J. Quinn T. J. (2020). Research with older people in a world with COVID-19: identification of current and future priorities, challenges and opportunities. *Age Ageing.* 10.1093/ageing/afaa149 [Epub ahead of print]. PMC7454250 32584954

[B78] Rita Egbert A. Cankurtaran S. Karpiak S. (2020). Brain abnormalities in COVID-19 acute/subacute phase: a rapid systematic review. *Brain Behav. Immun.* 10.1016/j.bbi.2020.07.014 [Epub ahead of print]. 32682993 PMC7366124

[B79] Rogers J. P. Chesney E. Oliver D. Pollak T. A. Mcguire P. Fusar-Poli P. (2020). Psychiatric and neuropsychiatric presentations associated with severe coronavirus infections: a systematic review and meta-analysis with comparison to the COVID-19 pandemic. *Lancet Psychiatry* 7 611–627. 10.1016/s2215-0366(20)30203-0 32437679 PMC7234781

[B80] Roman G. C. Spencer P. S. Reis J. Buguet A. Faris M. E. A. Katrak S. M. (2020). The neurology of COVID-19 revisited: a proposal from the environmental neurology specialty group of the world federation of neurology to implement international neurological registries. *J. Neurol. Sci.* 414:116884. 10.1016/j.jns.2020.116884 32464367 PMC7204734

[B81] Romero-Sanchez C. M. Diaz-Maroto I. Fernandez-Diaz E. Sanchez-Larsen A. Layos-Romero A. Garcia-Garcia J. (2020). Neurologic manifestations in hospitalized patients with COVID-19: the ALBACOVID registry. *Neurology.* 95 e1060–e1070. 10.1212/WNL.0000000000009937 32482845 PMC7668545

[B82] Sasannejad C. Ely E. W. Lahiri S. (2019). Long-term cognitive impairment after acute respiratory distress syndrome: a review of clinical impact and pathophysiological mechanisms. *Crit. Care* 23:352. 10.1186/s13054-019-2626-z PMC6852966 31718695

[B83] Solomon I. H. Normandin E. Bhattacharyya S. Mukerji S. S. Keller K. Ali A. S. (2020). Neuropathological features of Covid-19. *N. Engl. J. Med.* 383 989–992. 10.1056/NEJMc2019373 32530583 PMC7304421

[B84] Suzuki M. Hotta M. Nagase A. Yamamoto Y. Hirakawa N. Satake Y. (2020). The behavioral pattern of patients with frontotemporal dementia during the COVID-19 pandemic. *Int. Psychogeriatr.* 10.1017/S104161022000109X [Epub ahead of print]. 32517839 PMC7348213

[B85] Sy M. Kitazawa M. Medeiros R. Whitman L. Cheng D. Lane T. E. (2011). Inflammation induced by infection potentiates tau pathological features in transgenic mice. *Am. J. Pathol.* 178 2811–2822. 10.1016/j.ajpath.2011.02.012 21531375 PMC3124234

[B86] The Food and Drug Administration (2020). *FDA Guidance on Conduct of Clinical Trials of Medical Products DURING COVID-19 Public Health Emergency.* Available online at: https://www.fda.gov/regulatory-information/search-fda-guidance-documents/fda-guidance-conduct-clinical-trials-medical-products-during-covid-19-public-health-emergency (accessed July 24, 2020).

[B87] Troyer E. A. Kohn J. N. Hong S. (2020). Are we facing a crashing wave of neuropsychiatric sequelae of COVID-19? Neuropsychiatric symptoms and potential immunologic mechanisms. *Brain Behav. Immun.* 87 34–39. 10.1016/j.bbi.2020.04.027 32298803 PMC7152874

[B88] Tsai S. T. Lu M. K. San S. Tsai C. H. (2020). The neurologic manifestations of Coronavirus disease 2019 pandemic: a systemic review. *Front. Neurol.* 11:498. 10.3389/fneur.2020.00498 32574246 PMC7248254

[B89] University of Liverpool (2020). *COVID-19 Drug Interactions.* Available online at: https://www.covid19-druginteractions.org/ (accessed July 24, 2020).

[B90] Varatharaj A. Thomas N. Ellul M. A. Davies N. W. S. Pollak T. A. Tenorio E. L. (2020). Neurological and neuropsychiatric complications of COVID-19 in 153 patients: a UK-wide surveillance study. *Lancet Psychiatry* 7 875–882. 10.1016/S2215-0366(20)30287-X 32593341 PMC7316461

[B91] Vindegaard N. Benros M. E. (2020). COVID-19 pandemic and mental health consequences: systematic review of the current evidence. *Brain Behav. Immun.* 10.1016/j.bbi.2020.05.048 [Epub ahead of print]. 32485289 PMC7260522

[B92] Walker K. A. Gottesman R. F. Wu A. Knopman D. S. Gross A. L. Mosley T. H. (2019). Systemic inflammation during midlife and cognitive change over 20 years: the ARIC Study. *Neurology* 92 e1256–e1267. 30760633 10.1212/WNL.0000000000007094 PMC6511107

[B93] Ward C. F. Figiel G. S. Mcdonald W. M. (2020). Altered mental status as a novel initial clinical presentation for COVID-19 infection in the elderly. *Am. J. Geriatr. Psychiatry* 28 808–811. 10.1016/j.jagp.2020.05.013 32425470 PMC7227566

[B94] Weinberg M. S. Patrick R. E. Schwab N. A. Owoyemi P. May R. Mcmanus A. J. (2020). Clinical trials and tribulations in the COVID-19 era. *Am. J. Geriatr. Psychiatry* 28 913–920. 10.1016/j.jagp.2020.05.016 32507686 PMC7236727

[B95] Williamson E. J. Walker A. J. Bhaskaran K. Bacon S. Bates C. Morton C. E. (2020). OpenSAFELY: factors associated with COVID-19 death in 17 million patients. *Nature* 584 430–436. 32640463 10.1038/s41586-020-2521-4 PMC7611074

[B96] World Health Organization (2020). *Coronavirus Disease (COVID-19) Situation Report-190.* Available online at: https://www.who.int/docs/default-source/coronaviruse/situation-reports/20200928-weekly-epi-update.pdf?sfvrsn=9e354665_6 (accessed September 28, 2020).

[B97] Wu Z. McGoogan J. M. (2020). Characteristics of and important lessons from the Coronavirus disease 2019 (COVID-19) outbreak in China: summary of a report of 72314 cases from the Chinese center for disease control and prevention. *JAMA* 323 1239–1242. 10.1001/jama.2020.2648 32091533

[B98] Xia H. Lazartigues E. (2008). Angiotensin-converting enzyme 2 in the brain: properties and future directions. *J. Neurochem.* 107 1482–1494. 10.1111/j.1471-4159.2008.05723.x 19014390 PMC2667944

[B99] Yang Y. Shen C. Li J. Yuan J. Wei J. Huang F. (2020). Plasma IP-10 and MCP-3 levels are highly associated with disease severity and predict the progression of COVID-19. *J. Allergy Clin. Immunol.* 146 119–127.e4. 32360286 10.1016/j.jaci.2020.04.027 PMC7189843

[B100] Yuan B. Li W. Liu H. Cai X. Song S. Zhao J. (2020). Correlation between immune response and self-reported depression during convalescence from COVID-19. *Brain Behav. Immun.* 88 39–43. 10.1016/j.bbi.2020.05.062 32464158 PMC7247486

[B101] Zhang J. Lu H. Zeng H. Zhang S. Du Q. Jiang T. (2020). The differential psychological distress of populations affected by the COVID-19 pandemic. *Brain Behav. Immun.* 87 49–50. 10.1016/j.bbi.2020.04.031 32304883 PMC7156946

[B102] Zubair A. S. Mcalpine L. S. Gardin T. Farhadian S. Kuruvilla D. E. Spudich S. (2020). Neuropathogenesis and neurologic manifestations of the Coronaviruses in the age of Coronavirus disease 2019: a review. *JAMA Neurol.* 77 1018–1027. 10.1001/jamaneurol.2020.2065 32469387 PMC7484225

